# Analysis of salivary transcripts and antigens of the sand fly *Phlebotomus arabicus*

**DOI:** 10.1186/1471-2164-10-282

**Published:** 2009-06-25

**Authors:** Jitka Hostomská, Věra Volfová, Jianbing Mu, Mark Garfield, Iva Rohoušová, Petr Volf, Jesus G Valenzuela, Ryan C Jochim

**Affiliations:** 1 Charles University in Prague, Faculty of Science, Department of Parasitology, Vinicna 7, 128 44 Praha 2, Czech Republic; 2 Vector Molecular Biology Unit, Laboratory of Malaria and Vector Research, NIAID, NIH, Rockville, MD, 20852, USA; 3 Research Technologies Branch, NIAID, NIH, Rockville, MD, 20852, USA

## Abstract

**Background:**

Sand fly saliva plays an important role in blood feeding and *Leishmania *transmission as it was shown to increase parasite virulence. On the other hand, immunity to salivary components impedes the establishment of infection. Therefore, it is most desirable to gain a deeper insight into the composition of saliva in sand fly species which serve as vectors of various forms of leishmaniases. In the present work, we focused on *Phlebotomus (Adlerius) arabicus*, which was recently shown to transmit *Leishmania tropica*, the causative agent of cutaneous leishmaniasis in Israel.

**Results:**

A cDNA library from salivary glands of *P. arabicus *females was constructed and transcripts were sequenced and analyzed. The most abundant protein families identified were SP15-like proteins, ParSP25-like proteins, D7-related proteins, yellow-related proteins, PpSP32-like proteins, antigen 5-related proteins, and 34 kDa-like proteins. Sequences coding for apyrases, hyaluronidase and other putative secreted enzymes were also represented, including endonuclease, phospholipase, pyrophosphatase, amylase and trehalase. Mass spectrometry analysis confirmed the presence of 20 proteins predicted to be secreted in the salivary proteome. Humoral response of mice bitten by *P. arabicus *to salivary antigens was assessed and many salivary proteins were determined to be antigenic.

**Conclusion:**

This transcriptomic analysis of *P. arabicus *salivary glands is the first description of salivary proteins of a sand fly in the subgenus *Adlerius*. Proteomic analysis of *P. arabicus *salivary glands produced the most comprehensive account in a single sand fly species to date. Detailed information and phylogenetic relationships of the salivary proteins are provided, expanding the knowledge base of molecules that are likely important factors of sand fly-host and sand fly-*Leishmania *interactions. Enzymatic and immunological investigations further demonstrate the value of functional transcriptomics in advancing biological and epidemiological research that can impact leishmaniasis.

## Background

Phlebotomine sand flies are the arthropod vectors of *Leishmania *parasites, the causative agents of leishmaniasis. During the feeding process sand flies inject saliva into the site of the bite to facilitate successful acquisition of a blood meal [1]. An infected sand fly regurgitates infective metacyclic promastigote stage *Leishmania *while feeding; thus, the parasite is always introduced to the host as a mixture with sand fly saliva. Sand fly saliva facilitates the transmission of *Leishmania *parasites to mammalian hosts; at the same time, immune response to salivary components was shown to partially protect the host from *Leishmania *infection [2]. Therefore, salivary components essential for parasite transmission and/or eliciting protective immune response are sought-after. Salivary proteins from *Phlebotomus papatasi*, the vector of *Leishmania major*, and *Lutzomyia longipalpis*, the vector of *L. infantum*, have been extensively studied. In addition, cDNA libraries from several other sand fly species were characterized and include other sand flies that vector *L. major *(*P. duboscqi*), *L. infantum *(*P. ariasi *and *P. perniciosus*) and *L. donovani *(*P. argentipes*).

*Phlebotomus (Adlerius) arabicus *is distributed in certain parts of East Africa and the Middle East. In Ethiopia, *P. arabicus *females infected with uncharacterized *Leishmania *sp. were reported [3], and in northern Israel *P. arabicus *is the proven vector of cutaneous leishmaniasis caused by *L. tropica *[4,5]. Cutaneous leishmaniasis caused by *L. tropica *is found in a vast discontinuous area reaching from the south-western Mediterranean to Turkey, north-western India and Sub-Saharan Africa [6]. Long supposed to circulate in anthroponotic foci exclusively, *L. tropica *was recently shown to occur as an anthropozoonosis as well [4,5]. Laboratory experiments demonstrated that *P. arabicus *is a permissive vector, meaning it is susceptible to development of more than one species of *Leishmania*, including *L. major *and *L. infantum *[7].

In the present study, salivary gland transcripts and proteins of *P. (Adlerius) arabicus *were studied by cDNA sequencing, electrophoretic and proteomic methods. This is the first study of the repertoire of salivary molecules of a vector of *L. tropica *and it is the first report of the composition of salivary proteins in the subgenus *Adlerius*.

## Results and Discussion

### Sequencing of salivary gland cDNA library

A cDNA library was constructed from salivary glands of *Phlebotomus arabicus *females dissected one day after emergence. From this cDNA library, 1152 random transcripts were selected and sequenced, resulting in 985 high quality sequences. Sequences were clustered together based on sequence homology and produced 107 clusters and 288 sequences were assessed as singletons (only one sequence). The term cluster may refer to either singletons or multiple homologous sequences. Similar to other sand flies studied so far, the most abundant transcripts were those coding for putative secretory proteins and resulted in 74 clusters with an average number of 7.65 sequences per cluster. Predicted proteins containing retention signals for endoplasmic reticulum and/or transmembrane domains were not treated as putatively secreted. An example of such proteins is the translocon-associated protein complex, δ subunit (*PabSP91*; GenBank accession number FJ427208), which has homologs previously designated as 16 kDa or 16.1 kDa salivary protein in *P. ariasi *or *L. longipalpis*, respectively.

Members of 21 different families were found among putative secretory proteins. BLAST comparison of translated nucleotide sequences with the NR protein database showed that overall, high similarity was observed namely with salivary proteins of *L. infantum *vectors *P. (Larroussius) ariasi *and *P. (L.) perniciosus*. The expected values of these matches were highly significant at values lower than E^-60^. To a lesser extent, similarity to sequences of salivary proteins of *P. (Euphlebotomus) argentipes*, the vector of *L. donovani *in India, was also observed. These findings are fully in concert with the close evolutionary relationship of *Larroussius *and *Adlerius *subgenera reported by Aransay et al. [8].

Some of the protein families contained multiple members. The observed variability among individual protein family members might be explained by intraspecific polymorphism, as the tested sample was heterogeneous (a pool of salivary glands from 30 female sand flies). Nevertheless, analysis of genetic variation of SP15 salivary protein in *P. papatasi *brought strong evidence that SP-15 is a multicopy gene [9]. While individual intraspecific variability of sand fly salivary proteins awaits broader analysis, we propose that the multiple homologous transcripts within protein families observed in this *P. arabicus *salivary gland cDNA library may reflect gene duplication events or allelic variation.

Full-length sequences were obtained for most clusters coding for putatively secreted proteins. Only sequences containing a signal peptide and a polyA tail in the coding cDNA were considered full-length. Table 1 lists clusters for which full length sequences were obtained, including the name of the sequence, the best match to NCBI NR database, the predicted molecular weight (M_w_) and isoelectric point (pI) of the mature protein, and the GenBank accession number of the nucleotide coding sequence. The table also includes information on the presence of individual proteins in *P. arabicus *salivary proteome, as confirmed by Edman degradation and/or mass spectrometry. A more detailed description of the putative secreted proteins follows, starting with proteins encoded by the most abundant transcripts:

**Table 1:** Most abundant salivary protein transcripts from Phlebotomus arabicus

Cluster				Best match to NR protein database			pI	M_w_	GenBank accession #
	Sequence name	Protein detected	Seq per cluster	ACCN #	Organism	E value			
PabSP2	14 kDa salivary protein A	Y	70	61817267	*P. ariasi*	5E-062	9.1	14.2	FJ538111
PabSP20	D7-related salivary protein A	Y	50	74099915	*P. ariasi*	1E-108	9.22	26.9	FJ538107
PabSP15	27 kDa salivary protein similar to ParSP25	Y	49	61744161	*P. ariasi*	9E-092	5.04	26.7	FJ538100
PabSP26	yellow-related salivary protein	Y	29	61373243	*P. ariasi*	0.0	8.40	42.9	FJ410293
PabSP4	antigen 5-related protein	Y	28	61373238	*P. ariasi*	1E-133	9.27	31.1	FJ439532
PabSP14	25 kDa salivary protein A similar to ParSP25	Y	18	61744161	*P. ariasi*	3E-090	5.04	24.8	FJ538102
PabSP31	25 kDa salivary protein similar to ParSP02	Y	14	61817261	*P. ariasi*	7E-066	10.27	25.0	EZ000628
PabSP52	putative salivary phospholipase A2		14	76446615	*P. perniciosus*	1E-150	8.76	29.8	EZ000627
PabSP49	putative salivary endonuclease	Y	14	61807170	*P. ariasi*	0.0	9.45	40.5	FJ439531
PabSP53	46 kDa salivary protein		12	76446617	*P. perniciosus*	1E-172	5.97	46.4	FJ538106
PabSP34	34 kDa-like salivary protein B	Y	12	61807168	*P. ariasi*	1E-139	8.89	34.1	FJ489242
PabSP45	14 kDa salivary protein B	Y	12	61807162	*P. ariasi*	8E-060	9.33	14.2	FJ538112
PabSP30	26 kDa salivary protein similar to ParSP02	Y	12	61817261	*P. ariasi*	1E-068	10.32	26.3	EZ000629
PabSP39	salivary apyrase A	Y	11	61817259	*P. ariasi*	1E-167	8.89	35.3	EZ000633
PabSP56	2.7 kDa salivary peptide		9	61744159	*P. ariasi*	0.015	12.31	2.7	FJ538099
PabSP32	34 kDa-like salivary protein A	Y	8	61807168	*P. ariasi*	1E-160	8.89	34.0	FJ489241
PabSP29	28 kDa salivary protein similar to ParSP02		8	61817261	*P. ariasi*	4E-069	10.52	27.8	EZ000630
PabSP54	D7-related salivary protein C	Y	7	61744153	*P. ariasi*	1E-103	9.11	26.7	FJ538109
PabSP59	D7-related salivary protein B		7	61807164	*P. ariasi*	1E-102	9.42	27.4	FJ538108
PabSP16	25 kDa salivary protein B similar to ParSP25	Y	6	61744161	*P. ariasi*	1E-089	5.03	24.8	FJ538103
PabSP13	25 kDa salivary protein C similar to ParSP25	Y	5	61744161	*P. ariasi*	6E-091	5.04	24.9	FJ538104
PabSP72	salivary hyaluronidase		4	4887110	*Lu. longipalpis*	2E-096	9.07	53.0	FJ439533
PabSP41	salivary apyrase C	Y	4	61817259	*P. ariasi*	1E-168	8.89	35.3	EZ000631
PabSP75	10 kDa salivary protein similar to ParSP13		4	61744147	*P. ariasi*	2E-032	4.56	10.3	FJ474087
PabSP63	16 kDa salivary protein A		4	74486577	*P. argentipes*	4E-032	5.36	15.9	FJ474085
PabSP93	14 kDa salivary protein C		3	61807166	*P. ariasi*	4E-055	9.22	14.1	FJ538113
PabSP64	16 kDa salivary protein B		3	74486577	*P. argentipes*	6E-033	5.18	16.0	FJ474086
PabSP97	10 kDa salivary protein similar to ParSP21		3	61744157	*P. ariasi*	5E-50	5.11	10.3	FJ474088
PabSP12	25 kDa salivary protein D similar to ParSP25	Y	3	61744161	*P. ariasi*	9E-090	5.04	24.8	FJ538105
PabSP11	27 kDa salivary protein B similar to ParSP25	Y	3	61744161	*P. ariasi*	2E-090	4.97	26.6	FJ538101
PabSP40	salivary apyrase B	Y	3	61817259	*P. ariasi*	1E-168	8.77	35.3	EZ000632
PabSP84	D7-related salivary protein D		3	74099915	*P. argentipes*	1E-053	9.34	28.1	FJ538110
PabSP107	22 kDa salivary protein		2	94468382	*Ae. aegypti*	1E-27	4.37	22.2	EZ000635
PabSP126	17 kDa salivary protein		2	1.09E+08	*Ae. aegypti*	5E-049	5.34	17.2	EZ000636

Detection of a protein in the proteome that matches the predicted peptide sequence of the transcript is denoted under Protein detected by "Y"

### SP15-like proteins

**Figure 1:**
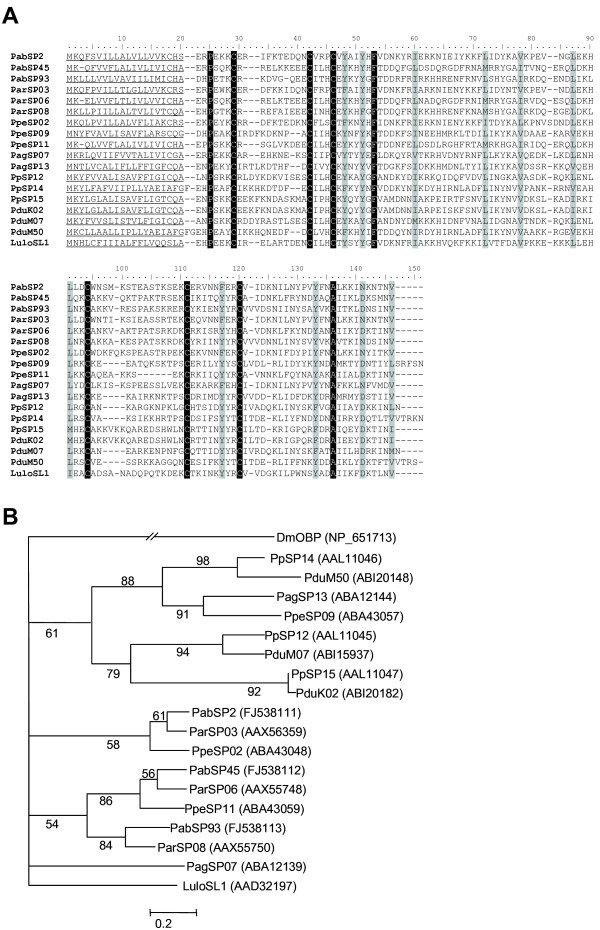
**Analysis of sand fly salivary proteins of the SP15 family**. (A) Multiple sequence alignment of SP15 and SP15-like salivary proteins from *Phlebotomus arabicus *(Pab), *P. ariasi *(Par), *P. argentipes *(Pag), *P. perniciosus *(Ppe), *P. papatasi *(Pp), *P. duboscqi *(Pdu), and *Lutzomyia longipalpis *(Lulo). The predicted signal secretion peptide is underlined. Identical amino acid residues are highlighted black and similar residues are highlighted grey. (B) Phylogenetic analysis of amino acid sequences of SP15 and SP15-like salivary proteins from sand flies and an oderant-binding protein of *Drosophila melanogaster *(DmOBP). Accession numbers are in parentheses and node values indicate branch support.

Thus far, SP15-like proteins have only been reported in sand flies and their function remains unknown. It was suggested that SP15-like proteins were derived from an ancestral odorant-binding protein and were closely related to short D7 proteins [10]. Immunization of mice with *P. papatasi *SP15 conferred partial protection against *L. major *infection [11]. Transcripts coding for these proteins represented the most abundant family in *P. arabicus *salivary gland cDNA library and clustered into three separate groups (*PabSP2*, *PabSP45 *and *PabSP93*; GenBank accession numbers FJ538111, FJ538112 and FJ538113, respectively). The amino acid sequences of mature proteins coded by *P. arabicus *transcripts share 22.5% amino acid identity and 23.3% amino acid similarity. When SP15-like proteins from other sand flies were added to the analysis, only the six cysteines and three other amino acids were conserved in the amino acid sequence of mature proteins (Figure 1A), reflecting the previously reported divergence among SP15-like proteins in sand flies [10]. In *L. longipalpis *a single SP15-like protein was found, SL1. In *P. arabicus *and other *Phlebotomus spp*. studied so far multiple members of the SP15 family are present. A phylogenetic analysis revealed three separate groups of *P. arabicus *SP15-like proteins, showing close relationships to *P. ariasi *proteins ParSP03, ParSP06 and Par08, respectively (Figure 1B). The predicted pI of all three *P. arabicus *SP15-like variants is highly basic (average pI = 9.22), corresponding to the fact that most *Phlebotomus spp*. sand fly salivary proteins have very high predicted pI values.

### 27 kDa and 25 kDa proteins

Six clusters coding for proteins related to *P. ariasi *27 kDa salivary protein (ParSP25; GenBank accession number AAX55664) and *P. perniciosus *29 kDa salivary protein (PpeSP08, GenBank accession number ABA43056) were found in the *P. arabicus *salivary gland cDNA library. There are no other homologs of these proteins in accessible databases, no conserved domains were found in the translated sequences, and no function has been assigned to these proteins. However, in *P. arabicus *cDNA library they represent the second most abundant protein family. Transcripts coding for ParSP25-like proteins occurred in long (*PabSP15 *and *PabSP11*; GenBank accession numbers FJ538100 and FJ538101) and short forms (*PabSP14*, *PabSP16*, *PabSP13 *and *PabSP12*; GenBank accession numbers FJ538102, FJ538103, FJ538104 and FJ538105, respectively), with very little variability among individual clusters. The mature proteins coded by these transcripts have a predicted M_w _of 27 kDa and 25 kDa, respectively, and are composed of 90.5% identical amino acids and 0.9% similar amino acids. The predicted pI of the proteins is acidic (average pI = 5.03), unlike most sand fly salivary proteins described thus far.

### D7-related proteins

**Figure 2:**
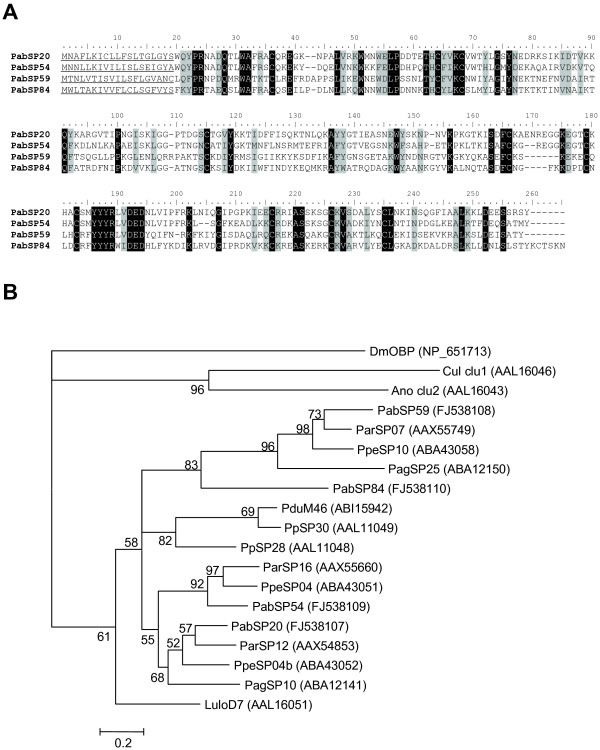
**Analysis of the D7-related family of sand fly and mosquito salivary proteins**. (A) Multiple sequence alignment of D7-related salivary proteins from *Phlebotomus arabicus *(Pab). The predicted signal secretion peptide is underlined. Identical amino acid residues are highlighted black and similar residues are highlighted grey. (B) Phylogenetic analysis of amino acids of D7-related proteins from *P. ariasi *(Par), *P. perniciosus *(Ppe), *P. argentipes *(Pag), *P. papatasi *(Pp), *P. duboscqi *(Pdu), and *Lutzomyia longipalpis *(Lulo), long form D7 proteins from mosquitoes *Culex quinquefasciatus *(Cul) and *Anopheles stephensi *(Ano), and odorant-binding protein from *Drosophila melanogaster *(DmOBP). Accession numbers are in parentheses and node values indicate branch support.

D7 proteins are well known from the saliva of mosquitoes, sand flies, black flies and biting midges [12-15]. While the structure of anopheline D7 proteins allows binding of biogenic amines and components of the contact activation system of coagulation [16,17], related proteins in sand flies lack conserved residues responsible for stabilizing bound ligands [18]. Thus, they may not interfere with host hemostasis by a similar mechanism and their function remains unknown. Mosquito D7 proteins elicit IgE in individuals hypersensitive to mosquito bites [19] and antibodies against sand fly D7 proteins were found in dogs naturally exposed to *L. longipalpis *[20]. Thus, it is possible that sand fly D7 proteins are involved in human hypersensitivity to sand fly bites. Four clusters of sequences coding for D7-related proteins were found in the *P. arabicus *salivary gland cDNA library (*PabSP20*, *PabSP59*, *PabSP54 *and *PabSP84*; GenBank accession numbers FJ538107, FJ538108, FJ538109 and FJ538110, respectively). Predicted mature proteins have M_w _of 26–28 kDa and an average basic pI of 9.24. Two of the seven clusters have potential N-glycosylation sites, as predicted by NetNGlyc server. The protein sequences of mature proteins were 20.3% identical and 15.5% similar (Figure 2A). The phylogenetic analysis showed four distinct clades among *P. arabicus *D7-related proteins, all of them bearing high similarity to *P. ariasi *and *P. perniciosus *proteins (Figure 2B).

### Yellow-related proteins

Yellow-related proteins are common in insects; in bloodsucking Diptera, yellow-related proteins were described from mosquitoes and sand flies. In *Ae. aegypti*, a dopachrome-converting enzyme shares homology with *Drosophila melanogaster *yellow proteins [21] and, according to Li et al. [22], it might play a role in melanotic encapsulation of parasites in the hemocoel. In sand fly salivary gland samples, however, dopachrome-converting enzyme activity could not be detected (Hostomska, unpublished observations), while yellow protein of *P. duboscqi *was detected in midgut and salivary glands and shown to have lectin properties [23]. Sand fly yellow proteins were previously proposed as potential antigens recognized by sera of experimentally bitten mice and dogs, and naturally exposed humans [24-26]. In *L. longipalpis *this was also suggested by mass spectrometry [20]. In the *P. arabicus *salivary gland cDNA library a single homolog of yellow-related proteins was found (*PabSP26*; GenBank accession number FJ410293). The predicted M_w _of the protein is 42.9 kDa with a pI of 8.4. No N-glycosylation sites were predicted in the protein sequence by amino acid submission to the NetNGlyc server.

### PpSP32-like proteins

**Figure 3:**
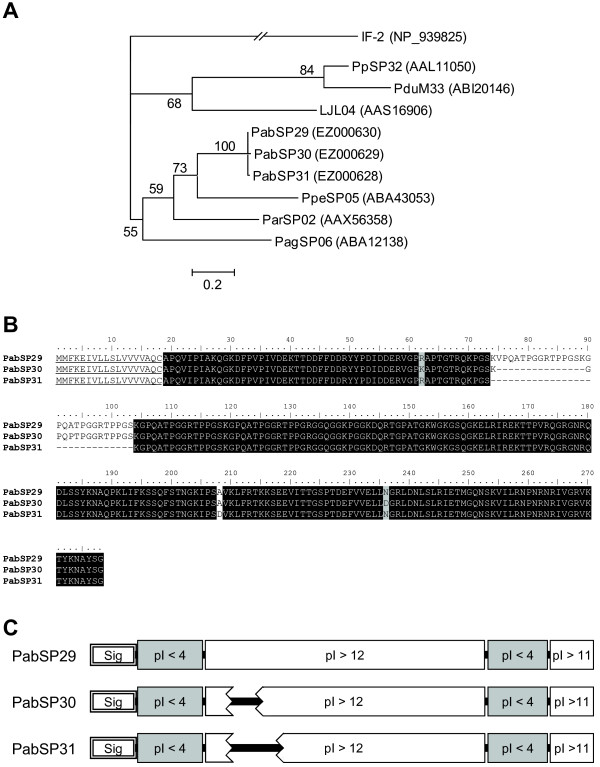
**Analysis of the PpSP32 family of sand fly salivary proteins**. (A) Phylogenetic analysis of amino acids of PpSP32-like salivary proteins from *Phlebotomus arabicus *(Pab), *P. ariasi *(Par), *P. perniciosus *(Ppe), *P. argentipes *(Pag), *P. papatasi *(Pp), *P. duboscqi *(Pdu), and *Lutzomyia longipalpis *(LJL), and translation initiation factor from *Corynebacterium diphteriae *(IF-2). (B) Multiple sequence alignment of PpSP32-like salivary proteins from *P. arabicus*. The sequences of predicted signal peptides are underlined. Background shading represents identical and similar amino acids; black and grey, respectively. (C) Schematic alignment of PpSP32-like salivary proteins from *P. arabicus*. The predicted signal secretion peptide (Sig) and isoelectric point (pI) of specific blocks of amino acids are shown with acidic regions shaded grey.

PpSP32-like proteins, so named due to homology with proteins described from *P. papatasi*, have only been found in sand flies and their function is unknown. In *P. perniciosus *they possess a collagen-related internal sequence [10]. In *P. arabicus*, however, these proteins bear no significant similarity to collagen; this feature is shared with PpSP32-like proteins of *P. papatasi*, *P. ariasi *or *P. argentipes*. Similarly to other protein families analyzed, the phylogenetic position of *P. arabicus *PpSP32-like proteins is close to that of *P. ariasi *and *P. perniciosus *homologs (Figure 3A). Three different transcript clusters coding for PpSP32-like proteins were found in the *P. arabicus *salivary gland cDNA library (*PabSP31*, *PabSP30 *and *PabSP29*; GenBank accession numbers EZ000628, EZ000629 and EZ000630, respectively), the mature proteins being 88.1% identical (Figure 3B). The variable length of the central glycine-rich region of the protein sequence results in three different variants of mature proteins. The predicted M_w _of the three variants are 25, 26.3 and 27.8 kDa. In all three variants of these proteins, there are alternating regions of very acidic (pI 4.0) and very basic (pI>11.5) amino acids (Figure 3C). As shown in figure 3C, the basic regions include the central glycine-rich sequence and the C-terminal basic tail. No N-glycosylation sites were predicted for these proteins by the NetNGlyc server.

### Antigen 5-related proteins

**Figure 4:**
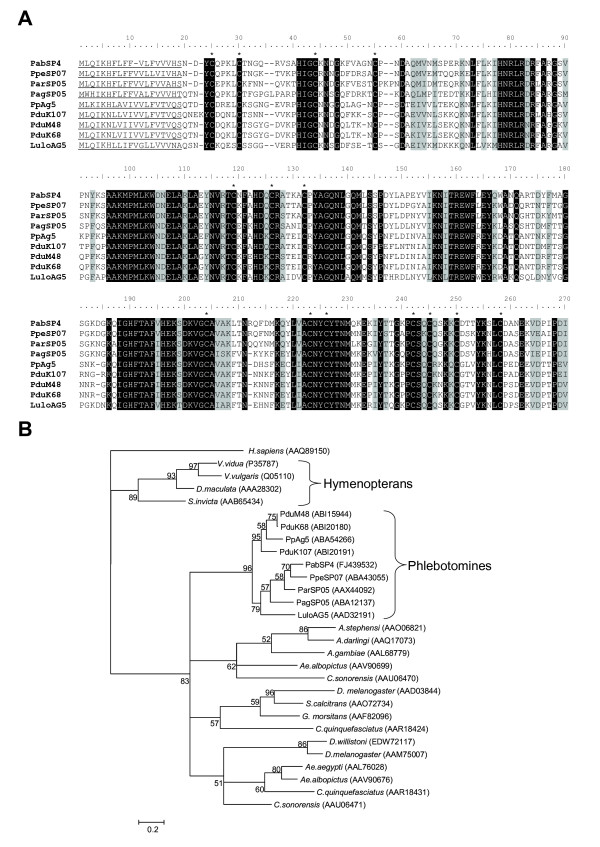
**Analysis of Ag 5 and Ag 5-related proteins**. (A) Multiple sequence alignment of Ag 5-related sand fly salivary proteins from *Phlebotomus arabicus *(Pab), *P. ariasi *(Par), *P. perniciosus *(Ppe), *P. argentipes *(Pag), *P. papatasi *(Pp), *P. duboscqi *(Pdu), and *Lutzomyia longipalpis *(Lulo). The sequences of predicted signal peptides are underlined. Background shading represents identical and similar amino acids; black and grey, respectively. Conserved cysteine residues are indicated by (*). (B) Phylogenetic analysis of Ag 5 and Ag 5-related protein amino acids from sand flies, *Aedes aegypti*, *Ae. albopictus*, *Culex quinquefasciatus*, *Anopheles gambiae*, *An. darlingi*, *An. stephensi*, *Culicoides sonorensis*, *Stomoxys calcitrans*, *Glossina morsitans morsitans*, *Drosophila melanogaster*, and *D. willistoni*, *Vespula vidua*, *V. vulgaris*, *Dolichovespula maculata*, and *Solenopsis invicta*, and a CRISP-family member from *Homo sapiens*. Accession numbers are in parentheses and node values indicate branch support.

Antigen 5 (Ag5) protein is present in vespid venom [27] and related proteins were reported in the saliva of bloodsucking insects [28,29]. Similar to most other sand fly species studied so far, only one cluster coding for Ag5-related protein was found in the *P. arabicus *cDNA library (*PabSP4*; GenBank accession number FJ439532) [10,28,30]. Mature Ag5-related proteins of sand flies are 45.6% identical and 14.5% similar, overall (Figure 4A). The phylogenetic analysis of Ag5-related proteins from sand flies, other blood-feeding insects and selected hymenopteran species shows a strongly supported distinct clade of sand fly Ag5-related proteins (Figure 4B). Unlike previous reports [10], this sand fly clade does not contain any *Culicoides *sequences. Close relationship of *P. arabicus *Ag5-related protein to *P. perniciosus *and *P. ariasi *was observed, much in the same way as in other salivary protein families (Figure 4B). The predicted M_w _of the mature protein is 31.1 kDa and the pI is very basic (9.27).

### Apyrase

Apyrases are widespread in saliva of bloodsucking insects. The antihemostatic effects of saliva are, for a great part, due to apyrase anti-platelet activity [31]. Sand fly apyrases belong to the *Cimex *apyrase family [32]. Three very similar apyrase clusters coding for apyrases were found in *P. arabicus *cDNA library (98.4% identity) (*PabSP41*, *PabSP40 *and *PabSP39*; GenBank accession numbers EZ000631, EZ000632 and EZ000633, respectively). The predicted average pI of *P. arabicus *apyrases is 8.85 and the predicted M_w _is 35.3 kDa.

### Endonuclease

A cluster encoding a putative endonuclease was identified in the *P. arabicus *cDNA library (*PabSP49*; GenBank accession number FJ439531). Similar sequences were reported from *P. ariasi*, *P. perniciosus*, *P. argentipes*, and *L. longipalpis *salivary glands. Cluster *PabSP49 *encodes an endonuclease domain, which is typical for DNA/RNA non-specific endonucleases. Since all residues composing the active site, the substrate binding site and the Mg^2+ ^binding site are conserved in this cluster; we suggest that PabSP49 might possess endonuclease activity. The predicted pI of the mature protein is 9.45 and the predicted M_w _of the mature protein is 40.5 kDa. Possible roles for a salivary endonuclease include reducing the viscosity of the blood pool during feeding and liberating nucleosides. Exogenous nucleosides, primarily adenosine, can exhibit regulatory effects on blood clotting, immune and inflammatory responses, and *Leishmania *pathogenesis [33].

### Hyaluronidase

**Figure 5:**
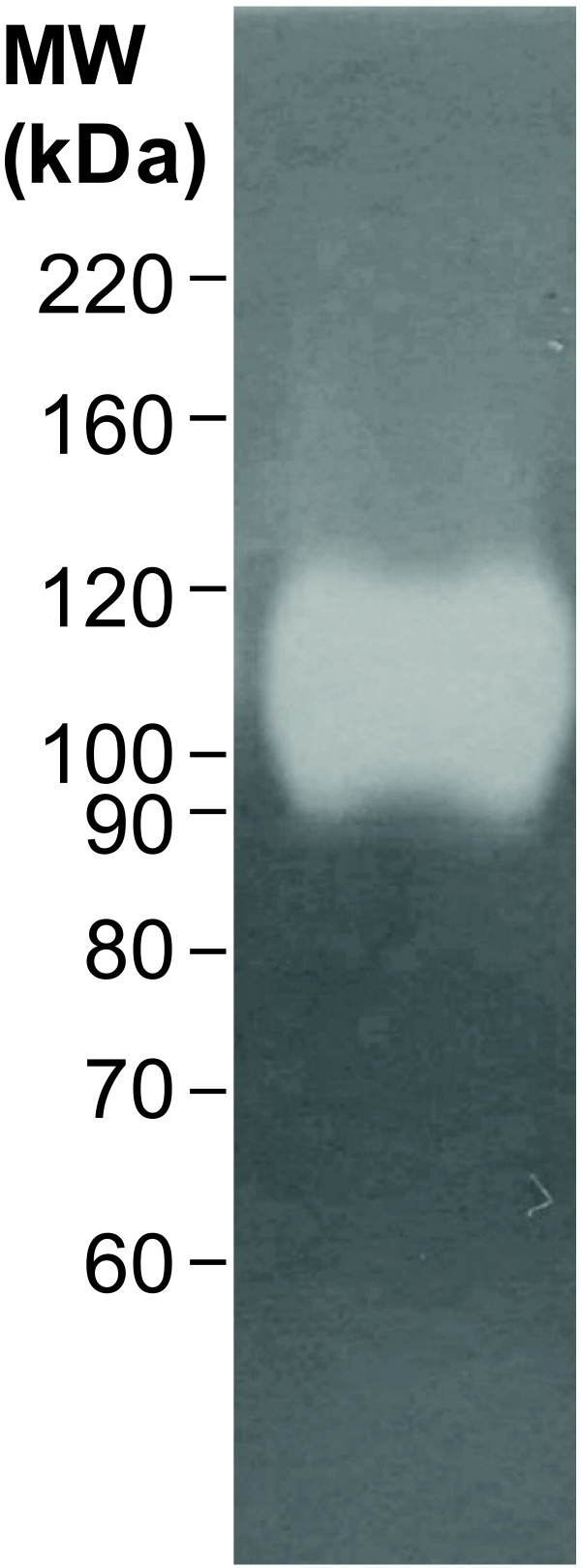
**Zymographic assay for hyaluronidase activity detection in salivary glands of *Phlebotomus arabicus***. Gels with incorporated hyaluronan were used for electrophoretic separation of salivary gland samples.

Hyaluronidase activity has been detected in several species of bloodsucking insects including sand flies [34,35]. Accessible expressed sequence tag (EST) data from cDNA libraries of *P. papatasi *and *P. duboscqi *salivary glands do not contain hyaluronidase transcripts. Nonetheless, the enzyme activity was detected in salivary gland samples from these species [34], highlighting the potent enzymatic activity of a protein produced from a low abundance transcript. In salivary gland homogenate of *P. arabicus*, hyaluronidase activity was also observed. As revealed by zymography, the apparent molecular weight of the *P. arabicus *hyaluronidase holoenzyme is approximately 110 kDa (Figure 5), but no protein band correspond to the predicted molecular weight could be detected by silver or Coomassie staining in electrophoretically separated salivary proteins. These observations reflect the scarcity of both hyaluronidase transcript and hyaluronidase protein in sand fly salivary glands, and at the same time underline the remarkably high specific activity of the enzyme. In *P. arabicus*, the predicted pI for mature hyaluronidase is 9.07 and the M_w _is 53 kDa.

### Additional putative enzymes

In the amino acid translation of sequence cluster 52 (*PabSP52*, GenBank accession number EZ000627), a phospholipase A2 (PLA2) domain is present, containing all conserved residues of both catalytic and metal-binding sites of PLA2. In hymenopteran venoms, PLA2 represents a major allergen. In the salivary glands of blood-feeding insects, sequences coding for PLA2-like proteins were reported only from sand flies of the subgenus *Larroussius *[10,30]. We tested salivary gland samples of *P. arabicus *specifically for PLA2 activity and did not detect any positive reaction. Cluster 52 contains an exceptionally long 5' untranslated region (5' UTR) compared to other clusters from this cDNA library coding for secreted proteins. The 5' UTR in this cluster is more than 500 nucleotides long. Thus, the regulation of expression of this transcript might be different from other transcripts reported herein.

Other sequences coding for other putative enzymes could not be obtained as full-length clones. These included a pyrophosphatase-like protein (*PabSP288*, GenBank accession number EZ000634), amylase (*PabSP47*, GenBank accession number EZ000626), an enzyme involved in digestion of dietary starch [36], and trehalase (*PabSP315*, GenBank accession number EZ000625). Previously, sequences coding for pyrophosphatase-like proteins were reported in *P. argentipes *and *P. duboscqi *sand flies [10,37]. These proteins, as well as their *P. arabicus *homolog reported herein, contain a conserved phosphodiesterase domain, typical for enzymes cleaving phosphodiester and phosphosulphate bonds in NAD, deoxynucleotides and nucleotide sugars [38]. Transcripts coding for α-amylase were found in *L. longipalpis *salivary glands and midguts as well as *P. papatasi *midguts [28,39,40]. Amylase activity was shown in *L. longipalpis *and *P. papatasi *salivary gland samples [28,36] and it is likely that the enzymatic activity is present also in *P. arabicus *salivary glands. The putative trehalase enzyme from *P. arabicus *salivary glands might either be an intrinsic component of insect metabolism, or might be related to sugar feeding and digestion. Trehalose is the main energy source in insect hemolymph in general. Trehalases are involved in its hydrolysis, yielding glucose molecules which are then readily available to various cells of the insect body [41]. So far, trehalase enzyme or sequence has not been reported from salivary glands of any blood-feeding insects, but sequences coding for sand fly trehalase have been found in midgut cDNA libraries of *P. papatasi *[40].

### Putative secreted proteins of unknown function

There were a number of transcripts with no homology to known enzymes or structural proteins; however, eight of these transcripts encode potentially secreted proteins with high homology to other sand fly salivary molecules. *P. arabicus *salivary transcripts code for 34 kDa proteins homologous to ParSP09. Polymorphisms resulting in different translations of the transcripts were observed (*PabSP32 *and *PabSP34*; GenBank accession numbers FJ489241 and FJ489242, respectively). These proteins are seemingly sand fly-specific; apart from 5 sand fly species (*P. ariasi*, *P. perniciosus*, *P. argentipes*, *P. duboscqi *and *L. longipalpis*); no other related proteins from any organism were reported. Another family of putative sand fly-specific proteins contain homologs of *P. arabicus *46 kDa salivary protein (*PabSP53*; GenBank accession number FJ538106). Transcripts coding for such proteins were reported from *P. perniciosus*, *P. ariasi*, *P. duboscqi *and *L. longipalpis*. Homologs of *P. arabicus *2.7 kDa peptide (*PabSP56*; GenBank accession number FJ538099) were only found in *P. ariasi *and *P. perniciosus *[10,30]. Our finding contradicts the suggestion that 2.7 kDa peptides are specific for the subgenus *Larroussius *[10]. Likewise, two unrelated 10 kDa proteins (*PabSP75 *and *PabSP97*; GenBank accession numbers FJ474087 and FJ474088, respectively) were found in *P. arabicus*; homologous molecules were identified in *P. ariasi *[30]. Additionally, two transcripts putatively encoding 16 kDa salivary proteins (two polymorphic variants *PabSP63 *and *PabSP64*; GenBank accession numbers FJ474085 and FJ474086, respectively) are homologous to molecules identified in *P. argentipes *[10].

Two sequence clusters coding for putatively secreted proteins in the *P. arabicus *cDNA library show no similarity with known sand fly sequences. Cluster 107 (*PabSP107*; GenBank accession number EZ000635) is homologous to *Ae. aegypti *putative salivary secreted mucin 3, as well as the IgE binding protein icarapin from honeybee venom [42]. The predicted molecular weight of the mature protein is 22.2 kDa and the putative protein would have an acidic pI of 4.4. There are 2 potential N-glycosylation sites and 9 potential O-glycosylation sites in the amino acid sequence of cluster 107, as predicted by submission to the NetNGlyc and NetOGlyc servers. Similarly, putative extracellular proteins of *Anopheles gambiae *(XP_001230739) and *Aedes aegypti *(XP_001650286) were also predicted to contain multiple O-glycosylation sites. These proteins might be involved in hypersensitivity to bites of blood-feeding insects. The second cluster 126 (*PabSP126*; GenBank accession number EZ000636), encodes a homolog of conserved hypothetical proteins of culicine as well as anopheline mosquitoes. The predicted molecular weight of the mature protein from *P. arabicus *is 17.2 kDa and the predicted pI is 5.34. No N- or O-glycosylation sites were predicted in cluster 126 protein and nothing is known about these hypothetical proteins.

### Proteome analysis of *P. arabicus *salivary glands

For the proteome analysis, *P. arabicus *salivary gland samples separated by SDS-PAGE were subjected to Edman degradation and mass spectrometry. Edman degradation resulted in the identification of 7 different N-terminal sequences. These were representative of two 14 kDa proteins (*PabSP2 *and *PabSP45*; GenBank accession numbers FJ538111 and FJ538112, respectively), yellow-related protein (*PabSP26*; FJ410293), and endonuclease (PabSP49; FJ439531). An N-terminal sequence common to all six variants of salivary proteins similar to ParSP25 was also detected by Edman degradation (*PabSP11-16*; GenBank accession numbers FJ538100–FJ538105), as well as N-terminal sequences common to apyrases (*PabSP39-41*; EZ000631-EZ000633) and to D7-related proteins A and C (*PabSP20 *and *PabSP54*; FJ538107 and FJ538109, respectively). From the data obtained by Edman degradation analysis it could not be concluded which variants of polymorphic salivary proteins were present in the proteome.

**Figure 6:**
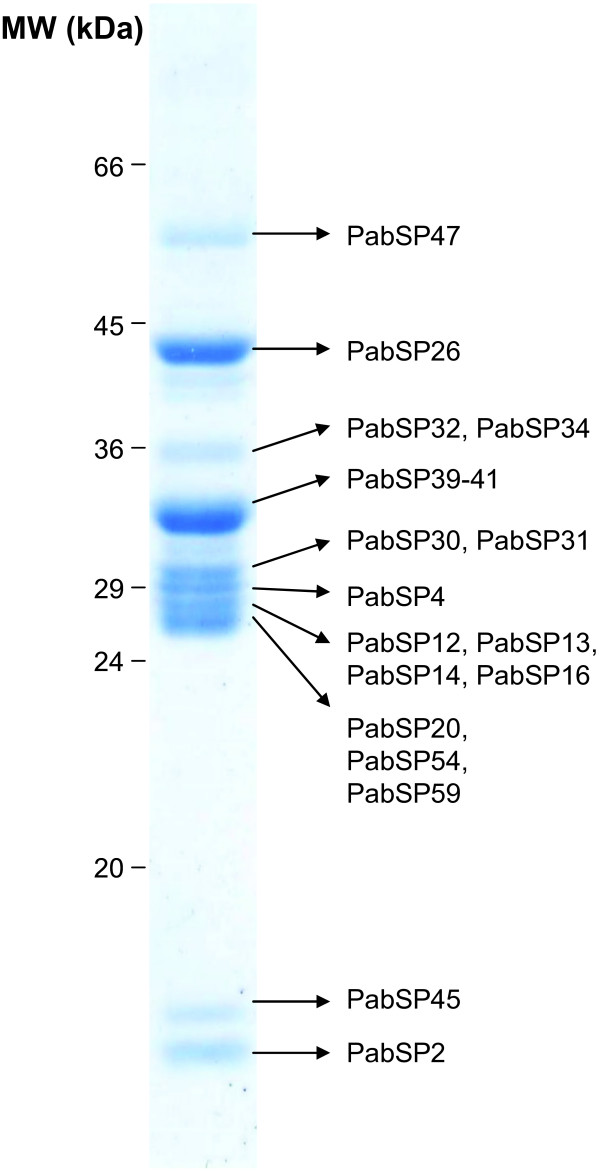
**Mass-spectrometric analysis of salivary gland proteins from *Phlebotomus arabicus***. Salivary gland samples were electrophoretically separated and individual bands cut from the Coomassie-stained gel were analyzed by mass spectrometry. GenBank accession number of the corresponding protein coding sequence is listed for each protein identified.

Mass spectrometry was used for a more detailed analysis of *P. arabicus *salivary proteome. By this method, 19 putative secreted proteins were identified in the proteome (Figure 6). These proteins include amylase (*PabSP47*, GenBank accession number ), yellow-related protein (*PabSP26*; GenBank accession number ), two 34 kDa salivary proteins (*PabSP32 *and *PabSP34*; GenBank accession number  and , respectively), all three apyrase-like proteins (*PabSP39*-*41*; GenBank accession number , , ), two PpSP32-like proteins (*PabSP31 *and *PabSP30*; GenBank accession number  and , respectively), antigen 5-related protein (*PabSP4*; GenBank accession number ), four 25 kDa salivary proteins similar to ParSP25 (*PabSP14*, *PabSP16*, *PabSP13 *and *PabSP12*; GenBank accession number , ,  and , respectively), three D7-related proteins (*PabSP20*, *PabSP59 *and *PabSP54*; GenBank accession number ,  and , respectively), and two PpSP15-like proteins (*PabSP2 *and *PabSP45*; GenBank accession number  and , respectively). In addition, one high-molecular weight protein (>70 kDa) analyzed by mass spectrometry revealed no similarity to predicted *P. arabicus *secreted salivary proteins. We assume this protein represents a component of salivary gland wall rather than a secreted protein present in the saliva. Accordingly, in *P. duboscqi *female salivary glands, we previously detected multiple protein bands running at molecular weight protein >70 kDa which were specifically present in the wall of salivary glands [43].

**Figure 7:**
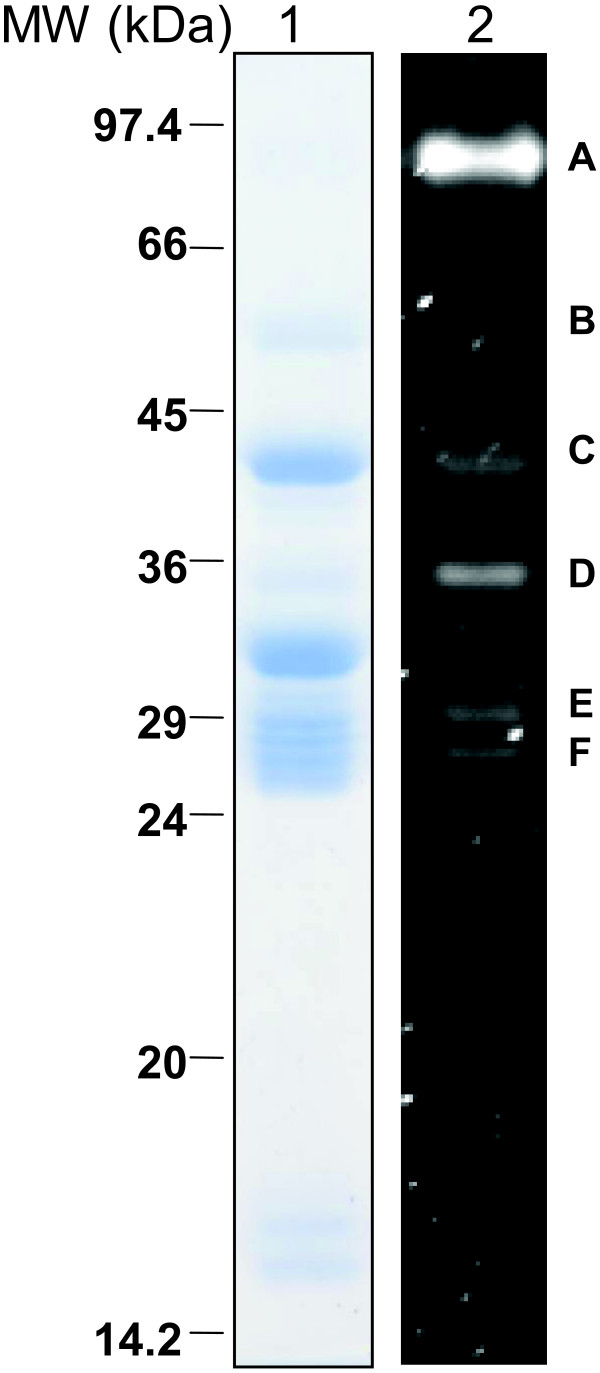
**Glycoprotein-specific staining of *Phlebotomus aribicus *salivary glands**. Salivary gland homogenate was electrophoretically separated in two wells of a polyacrylamide gel. The gel was cut in half with one portion stained with Coomassie (Lane 1) and the other portion stained with ProQ Emerald 300 Glycoprotein Stain (Lane 2). Six bands were visualized by glycoprotein staining (A-F).

Additionally, glycoprotein-specific staining of electrophoretically separated proteins was performed. ProQ Emerald staining detected six glycoprotein bands in *P. arabicus *salivary gland samples (Figure 7). Three bands (B, C and D) correlate with proteins identified by mass spectrometry: amylase (PabSP47), yellow-related protein (PabSP26), and 34 kDa proteins (PabSP32 and PabSP34). Band A is predicted to migrate at about 97 kDa and may represent hyaluronidase; however, this band may be produced by the oligomerization of other salivary proteins or components of the gland structure. Bands E and F do not distinctly correlate with molecules identified by mass spectrometry and are therefore unknown.

### Humoral response to *P. arabicus *saliva

**Figure 8:**
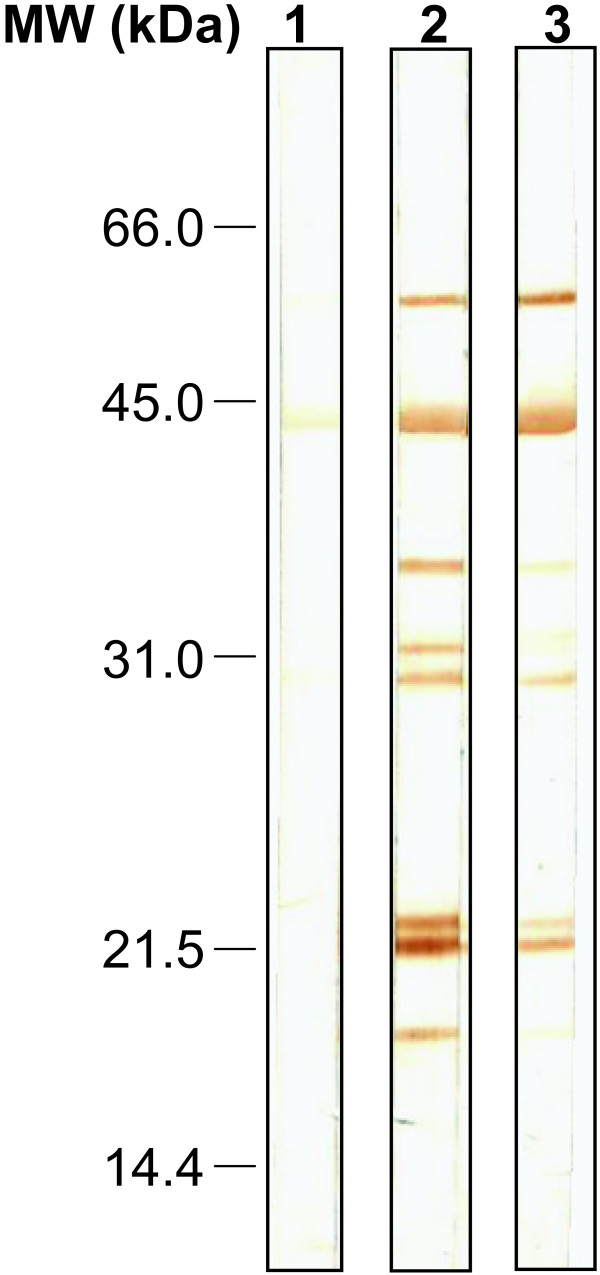
**Humoral response to salivary gland antigens of *Phlebotomus arabicus***. SDS-PAGE and immunoblotting of *P. arabicus *salivary gland proteins with sera of BALB/c mice: (1) non-immune serum; (2), (3) sera from mice repeatedly exposed to bites of *P. arabicus *females.

Some of the proteins homologous to *P. arabicus *salivary proteins are known as antigens or allergens in other insect species. *P. arabicus *salivary proteins elicit a strong antibody response in mice exposed to *P. arabicus *feeding. In Western blots, the most prominent antigenic bands recognized by sera of two bitten mice (Figure 8, lanes 2 and 3) had apparent molecular weights of 56–58.5 kDa, 45 kDa, 43 kDa (a double band), 42 kDa, 34.5–36.5 kDa and 30 kDa. Slightly weaker reactions were observed with bands running at 31 kDa and 30.5 kDa. In addition, sera from some animals recognized two very faint bands, running at 21 kDa and 16 kDa (Figure 8).

## Conclusion

In this study we generated a transcriptome of female sand fly *Phlebotomus arabicus *salivary glands using a PCR-based cDNA library. This is the first reported salivary gland transcriptome of a sand fly from the subgenus *Adlerius*. The most abundant transcripts were represented in the 985 high quality sequences. Many of the transcripts encoded full- or partial-length proteins; most of which are homologous to other sand fly species saliva molecules. Phylogenetic analysis consistently shows a strong relationship between *P. arabicus *with sand flies from the *Larroussius *subgenus; specifically, *P. ariasi *and *P. perniciousus*. The phylogenetic analyses of sand fly salivary proteins reaffirm the taxonomy of phlebotomines [7].

Some of the most abundant molecules identified in the transcriptome that have a predicted signal secretion peptide include a 14 kDa protein (PabSP2), a D7-related protein (PabSP20), a yellow-related protein (PabSP26), an Antigen 5-related protein (PabSP4) and 25 kDa and 27 kDa proteins similar to *P. ariasi *ParSP25 (PabSP14 and PabSP15, respectively). A number of paralogous transcripts were identified, such as those in the SP15 and D7 families. The presence of duplicate gene copies has been observed in other blood feeding arthropods [10,44] and can serve several potential functions including increased transcript abundance and rapid evolution of blood feeding strategies while retaining intrinsic proteins. Proteomic analysis by N-terminal sequencing or tryptic digestion followed by mass spectrometry identified 20 proteins in the salivary gland homogenate of *P. arabicus *that were characterized in the transcriptome. In addition, one protein was identified by mass spectrometry that did not match any of the characterized transcripts. This is the most comprehensive description of sand fly salivary proteome to date and also demonstrates that the transcriptome represents >95% of the most abundant proteins present in the salivary gland.

In the analysis of the *P. arabicus *salivary gland transcriptome four sequences were identified as encoding a putative hyaluronidase. Hyaluronidase is an enzyme that has been identified in a number of phlebotomine salivary glands including *Lutzomyia longipalpis*, *P. Phlebotomus papatasi*, *P. Phlebotomus duboscqi*, *P. Paraphlebotomus sergenti *and *P. Adlerius halepensis *[34]. The zymographic analysis of salivary gland extract confirms the presence of an active hyaluronidase enzyme and demonstrates the effectiveness of a transcriptomic approach to identifying disease vector salivary components.

Having described the repertoire of saliva molecules opens more doors in the research of vector-host and vector-parasite interactions, pharmacology and insect biochemistry. The antigenicity of sand fly saliva is one important aspect of the vector-host interaction. Evaluating the humoral response of mice bitten by *P. arabicus *demonstrates the abundance and diversity of antigenic molecules in the saliva. Future work may focus on the use of functional transcriptomics (expression of recombinant protein and biological and biochemical assays) to use these *P. arabicus *salivary proteins to evaluate the role of these molecules in the epidemiology of leishmaniasis.

## Methods

### Sand flies and salivary gland dissection

The colony of *P. arabicus *(Israel) was reared in the insectary of Charles University in Prague in standard conditions as described by Benkova and Volf [45]. For mRNA extraction salivary glands of 1-day-old females were dissected in saline and stored in RNA later (Ambion). For proteome analysis and Western blot analysis, salivary glands from 5- to 7-day-old *P. arabicus *females were dissected and stored in Tris buffer (20 mM Tris, 150 mM NaCl, pH 7.5).

### Construction of salivary gland cDNA library

Salivary gland mRNA was isolated from 30 pairs of glands using Micro-FastTrack mRNA isolation kit (Invitrogen). PCR-based cDNA library was made following the manufacturer's instructions for SMART™ cDNA library Construction Kit (BD Clontech) with some modifications described by Chmelar et al. [46]. The cDNA library was fractionated into three sets of cDNAs containing large, medium and small fragments. Gigapack^® ^III Gold Packaging Extract (Stratagene) was used for packaging the phage particles. The libraries were plated by infecting log-phase XL-1 blue *Escherichia coli *(Clontech). Several plaques from each plate were selected and a PCR with vector primers flanking the inserted cDNA was performed. The presence of recombinants was checked by visualisation the PCR products on 1.1% agarose gel with ethidium bromide.

### Sequencing of Selected cDNA Clones

Plaques were randomly selected from the plated libraries and transferred to 96-well polypropylene plate containing 75 μl of water per well. The PCR reaction amplifying randomly selected cDNAs was performed using FastStart PCR Master mix (Roche), 3 μl of the phage sample as a template and primers described elsewhere [30]. Amplification conditions were as follows: 1 hold of 75°C for 3 min, 1 hold of 94°C for 2 min and 34 cycles of 94°C for 1 min, 49°C for 1 min and 72°C for 2 min. Final elongation step lasted for 10 min at 72°C. Reaction products were cleaned using ExcelaPure 96-Well UF PCR Purification Plates (EdgeBio) and used as templates for cycle-sequencing reaction using BigDye Terminator v3.1 cycle sequencing kit (Applied Biosystems) and a forward primer described elsewhere [30]. Cycle-sequencing reaction products were cleaned using sephadex and MultiScreen HV Plates (Millipore), dried and stored at -20°C. Sequencing was performed on an ABI 3730Xl DNA sequencer (Applied Biosystems).

### Bioinformatics

Detailed description of the bioinformatic treatment of the data can be found elsewhere [29,46]. Briefly, EST trace files were analyzed using a customized program based on the Phred algorithm [47,48]. Sequences with Phred quality scores lower than 20 were removed, as well as primer and vector sequences. Resulting sequences were grouped into clusters using a customized program based on identity (95% identity, 64 word size) and aligned into contiguous sequences (contigs) using the CAP3 sequence assembly program [49]. BLASTX, BLASTN or RPS BLAST programs [50] were used to compare contigs and singletons (contigs with a single sequence) to the non-redundant (NR) protein database of the NCBI, the gene ontology (GO) fasta subset [51], to the conserved domains database (CDD) of NCBI [52] which contains KOG ()[53], Pfam [54] and Smart databases[55], and to mitochondrial and rRNA nucleotide sequences available from NCBI. The three frame translations of each dataset were submitted to the SignalP server [56] to detect signal peptides. The grouped and assembled sequences, BLAST results and SignalP results were combined in an Excel spreadsheet and manually verified and annotated. N- and O-glycosylation site prediction was performed for selected sequences using NetNGlyc 1.0 and NetOGlyc 3.1 software (www.cbs.dtu.dk/services/NetNGlyc, www.cbs.dtu.dk/services/NetOGlyc) [57].

### Phylogenetic analysis

Protein sequences of the members of identified protein families were compared with related sequences of other sand fly species obtained from GenBank. Sequences were aligned using ClustalW version 1.4 [58] running under BioEdit sequence-editing software, version 7, and manually refined in BioEdit. For each alignment, best substitution matrix was determined by ProtTest software, version 1.4 [59]. This matrix was then used by TREE-PUZZLE 5.2 [60] to reconstruct phylogenetic trees from the protein alignments by maximum likelihood. TREE-PUZZLE implements quartet puzzling (QP) tree search; at the same time, the algorithm estimates support values for each internal branch. The number of puzzling steps was 1000 in each phylogenetic analysis. Resulting trees were viewed in MEGA 4 [61].

### Proteome analysis

Salivary glands from 5-day-old *P. arabicus *females were homogenized by 5 freeze-thaw cycles. Samples were reduced using sample buffer with 2-mercaptoethanol, and electrophoretically separated in 12% polyacrylamide SDS gel. Gels were stained for total proteins with Coomassie G-250 (SimplyBlue SafeStain, Invitrogen) or for glycoproteins with Pro-Q Emerald 300 glycoprotein stain (Invitrogen). Mass spectrometric analysis was performed with individual bands cut from the Coomassie-stained gel. The individual bands were placed in microtubes and covered with 100 μl 50 mM ammonium bicarbonate (ABC) buffer in 50% acetonitrile (ACN) with 50 mM dithiothreitol (DTT). The samples were subjected to sonication in an ultrasonic bath for 5 minutes. After 15 minutes the supernatant was discarded and the gel was covered with 100 μl of 50 mM ABC/50% ACN with 50 mM iodoacetamide and sonicated for 5 minutes. After 25 minutes, the supernatant was discarded and exchanged for 100 μl 50 mM ABC/50% ACN with 50 mM DTT and sonicated for 5 minutes to remove any excess iodoacetamide. The supernatant was discarded and samples were sonicated for 5 minutes in 100 μl of HPLC water. The water was discarded and samples were sonicated for another 5 minutes in 100 μl of ACN. ACN was discarded and microtubes with samples were left open for a couple of minutes to allow the rest of ACN to evaporate. Five ng of trypsin (Promega) in 10 μl of 50 mM ABC were added to the gel. Samples were incubated at 37°C overnight. Trifluoroacetic acid (TFA) and ACN were added to reach final concentration 1% TFA, 30% ACN. Samples were sonicated for 10 minutes and 0.5 μl drop was transferred onto MALDI target and let to dry. Dried droplets were covered with 0.5 μl drop of alpha-cyano-hydroxycinnamic acid solution (2 mg/ml in 80% ACN) and let to dry. Samples were measured using a 4800 Plus MALDI TOF/TOF analyzer (Applied Biosystems/MDS Sciex) equipped with a Nd:YAG laser (355 nm, firing rate 200 Hz).

Peak lists from the MS spectra were generated by 4000 Series Explorer V 3.5.3 (Applied Biosystems/MDS Sciex) without smoothing, peaks with local signal to noise ratio greater than 5 were picked and searched by local Mascot v. 2.1 (Matrix Science) against a database of proteins sequences derived from cDNA library. Database search criteria were as follows – enzyme: trypsin, taxonomy: none, fixed modification: carbamidomethylation, variable modification: methionine oxidation, peptide mass tolerance: 120 ppm, one missed cleavage allowed. Only hits that were scored as significant (p < 0.0001) are included.

For the Edman degradation analysis, *Phlebotomus arabicus *salivary glands were electrophoretically separated on 1 mm thick 4–20% NuPAGE Novex Bis-Tris gels using MES SDS buffer (Invitrogen). A sample containing 30 glands was reduced with NuPAGE Sample Reducing Agent (Invitrogen) and run in parallel with non-reduced samples (50 glands) on the same gel. Wet blotting on a PVDF membrane was performed using XCell II™ Blot Module (Invitrogen). SeeBlue^® ^Pre-Stained Standards (Invitrogen) were used to estimate molecular weight (M_w_) of separated proteins and assess transfer efficiency. The membrane was stained with 0.025% Coomassie blue without acetic acid. Stained bands were cut and subjected to Edman degradation using a Procise 494cLC sequencer (Applied Biosystems). cDNA sequences corresponding to obtained N-terminal amino acid sequences of salivary proteins were identified using an in-house search program [29]. This program compared three possible translations of each cDNA sequence obtained in the *P. arabicus *cDNA sequencing project with the amino acid sequences.

### Enzymatic assays

Salivary gland samples from 5-day-old *P. arabicus *females were tested for the activities of hyaluronidase and phospholipase A2. Salivary glands were dissected in Tris buffer (20 mM Tris, 150 mM NaCl, pH 7.8) and stored at -20°C. Before use, the glands were mechanically disrupted, samples were centrifuged at 12000 g for 5 minutes, and the supernatant was used in the assays.

The zymographic hyaluronidase assay was performed as described by Volfova et al. [35]. Briefly, salivary gland samples were separated by SDS electrophoresis in 8% polyacrylamide slab gels with incorporated hyaluronan (0.002%). Prior to electrophoresis, aliquots of the sample were subjected to treatment with reducing agents (dithiothreitol 50 mM, 45 minutes at 25°C, or 2-mercaptoethanol 7 mM, 45 minutes at 40°C). An equivalent of 1 salivary gland per lane was used for electrophoresis under reducing conditions using dithiothreitol, an equivalent of 2 salivary glands per lane for electrophoresis under reducing conditions using 2-mercaptoethanol, and for electrophoresis under non-reducing conditions, an equivalent of 1/12 of a gland per lane was used. After the electrophoresis, SDS was washed out of the gels; the gels were equilibrated with 0.1 M acetate buffer and incubated at 37°C for 120 minutes. Gels stained with Stains-all (Sigma) were rinsed in distilled water and documented.

The phospholipase A2 activity was tested in the same salivary gland samples using the EnzChek^® ^Phospholipase A_2 _Assay Kit (Invitrogen). The manufacturer's instructions were followed. Samples containing 0.5, 1, 2, 5, 10 and 20 salivary glands in 50 μl were assayed; as a positive control, PLA2 from honey bee venom supplied with the kit was used. The assay was performed at 26°C, and fluorescence emission at 515 nm was detected after an incubation step of 12 minutes (Tecan Infinite 200).

### Immunization of mice

Experiments on mice were done in accordance with Czech Act No. 246/1992 and approved by IACUC of the Faculty of Science, Charles University in Prague. Female BALB/c mice, 8 weeks old (Charles River Deutschland, Sulzfeld, Germany) were used for the exposure to *P. arabicus *sand flies. In biweekly intervals, the mice were anaesthetized with ketamine (1.5 mg/10 g body weight) and xylazin (0.15 mg/10 g body weight) and exposed to sand fly females. Each time, an average of 60 females fed on each mouse (SE = 14.9). Ten days after the sixth exposure the mice were bled and obtained sera were kept at -20°C.

### Western blotting

*Phlebotomus arabicus *salivary gland proteins were disrupted by three freeze-thaw cycles in liquid nitrogen and separated by SDS-PAGE on 12% polyacrylamide gel, 0.75 mm thick using Mini-Protean III apparatus (BioRad). As a reducing agent, 2.5% 2-mercaptoethanol was used in sample buffer. Biotinylated low range standards (BioRad) were run on the same gel. Separated proteins were electro-transferred onto a nitrocellulose membrane by iBlot™ Gel Transfer Device (Invitrogen), and the membrane was cut into strips corresponding to sample load of 5 salivary glands per strip. These strips carrying salivary gland samples were either stained with amidoblack (Merck; 0.1% solution in 25% isopropanol and 10% acetic acid) or blocked with 5% BSA in phosphate-buffered saline, pH 7.4, with 0.05% Tween-20 (PBS-Tw) overnight. The sample-carrying strips were incubated for 1 hour with pre-immune or immune sera of BALB/c mice exposed to *P. arabicus *(diluted 1:250 in PBS-Tw), and then with horseradish peroxidase-conjugated swine anti-mouse antibody (Sevapharma, 1:1000 in PBS-Tw) for 45 minutes. Streptavidin-conjugated peroxidase (Sigma, 1 μg/ml in PBS-Tw) was used for the biotinylated standards. The colour reaction was developed using H_2_O_2 _and diaminobenzidine in PBS. Washes between individual steps were done with PBS-Tw.

## Authors' contributions

JH participated in the cDNA library construction and annotation, sequence alignment and phylogenetic analysis, immunization of mice by sand flies and the detection of humoral response to sand fly saliva, and drafting the manuscript. JM sequenced all cDNA clones selected from the library. VV dissected the salivary glands and carried out the enzymatic assays. IR participated in sample preparation for Edman degradation, which was carried out by MG. PV and JGV conceived the study, participated in its design and coordination and revised the manuscript. RCJ carried out the bioinformatic analysis of transcript sequences, participated in coordination of the study and drafting the manuscript. All authors have read and approved the final manuscript.
